# The last bacterial common ancestor encoded a complex flagellum

**DOI:** 10.64898/2026.06.11.731707

**Published:** 2026-06-12

**Authors:** Berkay Selçuk, Ekaterina P. Andrianova, Morgan Beeby, Daniel B. Kearns, Marc Erhardt, Igor B. Zhulin

**Affiliations:** 1 Biophysics Graduate Program, The Ohio State University, Columbus, Ohio, USA; 2 Department of Microbiology, The Ohio State University, Columbus, Ohio, USA; 3 Translational Data Analytics Institute, The Ohio State University, Columbus, Ohio, USA; 4 Department of Life Sciences, Imperial College London, London, UK; 5 Department of Biology, Indiana University, Indiana, USA; 6 Humboldt-Universität zu Berlin, Institute of Biology, Berlin, Germany.; 7 Max Planck Unit for the Science of Pathogens, Berlin, Germany.

## Abstract

Bacterial flagella are rotary nanomachines that enable motility in diverse environments. Although more than 40 genes are required to assemble, operate, and regulate a functional flagellum in model organisms, only 24 flagellar genes have previously been inferred to be conserved across bacteria. This discrepancy raises a fundamental question: did the last bacterial common ancestor encode a simpler, partial flagellum that was elaborated later in a lineage-specific manner, or does the apparent absence of conserved components reflect limitations in detecting highly diverged homologs? Here we combine large-scale profile- and sequence-based searches across a comprehensive bacterial genome set with conserved sequence signatures, gene-tree clustering and flagellar gene-neighborhood evidence to reconstruct the ancestral complexity of bacterial flagellar systems. We identify 28 additional flagellar gene families whose distributions and evolutionary histories support an origin before major bacterial diversification, yielding a 52-gene ancestral flagellum. The ancestral flagellum included all proteins of the secretion/export apparatus, basal body, axial components, motor-force generators and regulatory checkpoints required to build and operate a functional, contemporary flagellum. These findings revise models of early bacterial evolution and overturn the notion that the ancestral flagellum was genetically minimal. Instead, they suggest that the last bacterial common ancestor possessed a highly complex flagellar system comprising more components than are typically found in extant bacteria, many of whose flagella appear to have been shaped by lineage-specific gene loss.

## INTRODUCTION

The bacterial flagellum is one of the best-studied molecular machines ^1,2^. It drives motility and chemotaxis ^3^, influences colony formation ^4^ and biofilm development ^5^, and plays key roles in pathogenicity ^6^ and host colonization ^7,8^. Flagellar motility is widespread among bacteria ^9^, and despite its high energetic cost, flagella are retained across diverse lineages, indicating a substantial contribution to fitness. In model organisms, it has been characterized in exceptional detail at the genetic, structural, and functional levels. Its biogenesis and assembly are tightly regulated, with components added in a precise temporal and spatial order, and it is integrated with complex signal transduction pathways ^10–12^. Owing to this combination of structural complexity and multi-step regulation, the flagellum is often cited as a model for understanding complex molecular systems ^13^. As such, it provides a powerful framework for investigating how complex molecular machines evolve, which remains a central question in biology.

The flagellum is composed of dozens of proteins organized into substructures, including basal body, hook, filament, and export apparatus, combing to form a functional rotary nanomachine. First, the export apparatus self-assembles into the inner membrane. A chassis structure (The “MS-ring”) assembles around the export apparatus and serves as the foundation for assembly of a cytoplasmic rotor ring (the “C-ring”) and periplasmic driveshaft (the rod”). The rod and subsequent axial structures assemble from subunits exported through the export apparatus powered by both proton motive force and ATP hydrolysis. Subsequently, proteins forming channels through the peptidoglycan and outer membrane assemble around the rod, allowing it to extend beyond the cell envelope. A short universal joint (the “hook”) then assembles at the rod tip. When the hook reaches its characteristic length, a molecular ruler triggers a specificity switch in the export apparatus, resulting in the export of filament proteins that assemble into a flagellum up to 10 μm long. Finally, a ring of motor protein complexes (the “stator complexes”) assemble in the inner membrane above the C-ring; proton flux through these stator complexes exerts torque on the C-ring and rotation of the flagellum via the MS-ring. In many species, the stator complexes are scaffolded to form a wider ring by additional scaffold structures. Regulation is facilitated by a host of non-structural proteins involved in transcriptional regulation and that chaperone assembly.

The prevailing view is that many bacteria share a conserved core set of flagellar genes alongside lineage-specific components ^14^. Previous studies identified a set of 24 “core” flagellar genes that were likely present in the last bacterial common ancestor (LBCA)^14,15^. More recent rooting of the bacterial tree and reconstruction of the bacterial ancestor further verified that the LBCA was flagellated ^16^, without explicitly defining a core gene set. However, experimentally studied flagellar systems in modern bacteria typically require more than 40 genes for the assembly, function and regulation of the flagellum ^11 17,18^. Consequently, the composition and complexity of the ancestral flagellum remain unresolved, in part because highly diverged homologs are difficult to detect across deep evolutionary timescales ^15,19,20^. Did LBCA encode a simpler, partial flagellum that was elaborated over time, or does the apparent absence of components reflect limitations in detecting diverged homologs? Here, using a comprehensive computational framework for homolog detection, we reconstruct the flagellar gene repertoire of the LBCA and show that it was substantially more complete than previously recognized, closely resembling those of extant bacteria and indicating that a functionally complete flagellum was already present at the earliest stages of bacterial evolution.

## RESULTS

### Defining a non-redundant flagellar protein dataset

To compile a comprehensive set of flagellum-associated proteins, we used the KEGG pathway map02040 (“Flagellar assembly”), which lists 54 proteins, as an initial reference set. To identify additional conserved flagellum-associated genes that may not be represented in KEGG, we compiled a representative set of 236 bacterial genomes that contain flagellar genes selected for broad taxonomic coverage, genome quality, including well-studied model organisms such as *Escherichia coli, Salmonella enterica* sv. Typhimurium, *Campylobacter jejuni*, *Helicobacter pylori*, *Borrelia burgdorferi* and *Bacillus subtilis* and searched against these genomes using profile Hidden Markov Models (HMMs) for each of the 54 flagellar proteins (see Methods for details). Distribution of identified flagellar proteins across 248 genomes is shown in Table S1. Inspection of the resulting flagellar operons identified several additional genes that were consistently associated with flagellar loci across diverse bacteria (*e.g*. *yvyF* and *yviE)* and the corresponding proteins were added to the set of flagellum-associated proteins. We further expanded this set through an extensive literature survey focused primarily on experimentally characterized genes in model organisms. Together, these approaches yielded an initial list of 95 proteins involved in the assembly, function, and regulation of flagella in diverse bacterial species. Following curation, synonymous gene names and homologous proteins representing the same functional group were consolidated into unified orthologous groups (Table 1). The final non-redundant dataset contained 81 orthologous groups of known and putative flagellum-associated proteins (Table 2), which served as the reference set for all downstream large-scale homology searches.

### Identifying flagellar proteins across the bacterial tree of life

**Figure 1:**
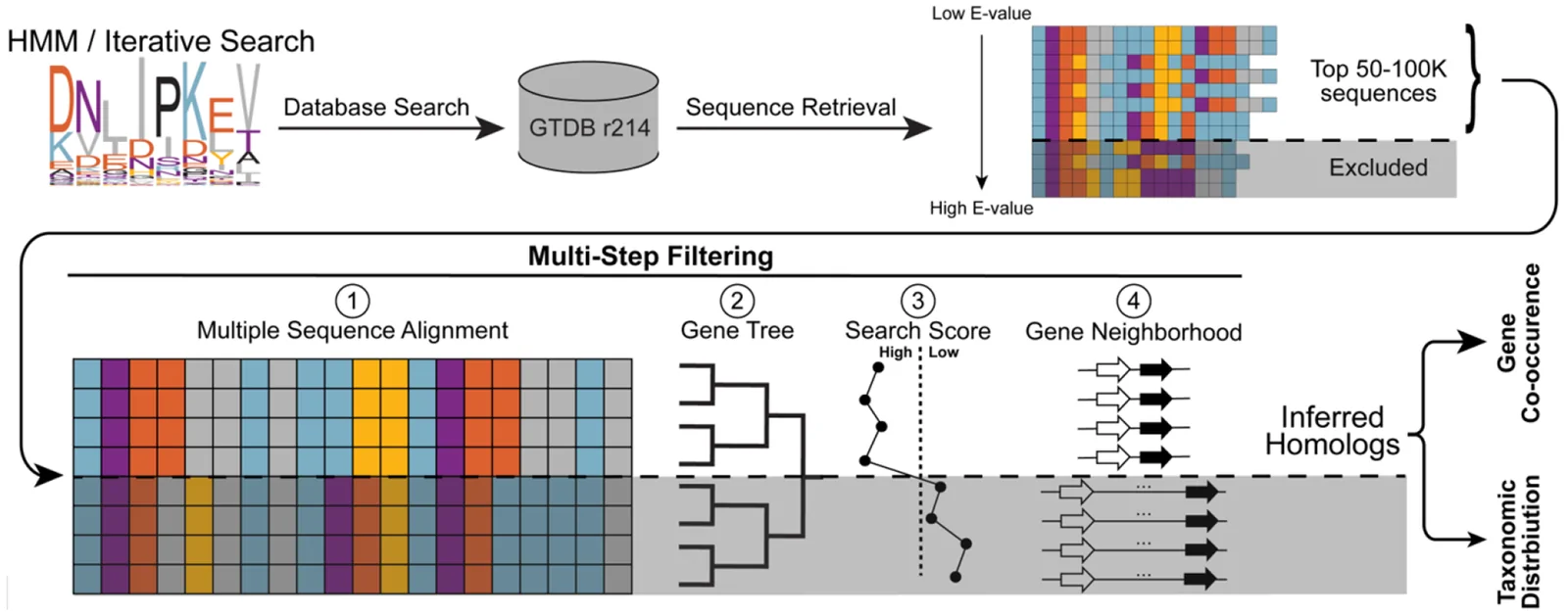
Computational framework for identifying flagellar proteins across Bacteria. For each flagellar protein corresponding HMMs or representative protein sequences were used to search for homologous sequences at GTDB r214 database. The top 50-100K hits, ranked by their E-value, were retrieved, aligned, and used to construct gene trees. Homologs of each flagellar protein were then inferred using four complementary criteria: conserved sequence patterns in multiple sequence alignments, clustering within gene trees, higher search scores, and co-localization with other flagellar genes within the same genomic neighborhood.

To identify flagellar proteins across bacteria, we developed a computational framework (Fig. 1) for sensitive detection of homologs across GTDB release 214 ^21^, comprising 80,789 representative bacterial proteomes. For each flagellar protein in the curated search set, we used Hidden Markov Models (HMMs) from the KOfamKOALA ^22^ and Pfam ^23^ databases where available, and representative annotated protein sequences as queries for iterative profile searches otherwise. Searches were performed using relaxed E-value thresholds to maximize sensitivity and capture divergent homologs. For most proteins, we retained the top 50,000 hits, a threshold chosen to capture potential flagellar homologs across the dataset (given that fewer than half of the ~80,000 genomes were expected to encode flagella^20^), while keeping searches and downstream analyses computationally tractable. For multicopy proteins such as FliC, MotA, and MotB, we retained the top 100,000 hits.

Sequences within clades satisfying these criteria were retained as inferred homologs for downstream analyses. Importantly, sequence similarity scores, conserved alignment patterns, and genomic neighborhood information were used to identify homologous clades rather than to evaluate individual sequences independently. Consequently, sequences with lower HMM scores, atypical sequence features, or no detectable association with flagellar gene neighborhoods could still be retained if they grouped within a well-supported homologous clade whose membership was supported by the combined evidence. This strategy increased sensitivity while maintaining confidence in homology assignments.

### Co-presence analysis reveals conserved and functionally coherent flagellar gene clusters

**Figure 2:**
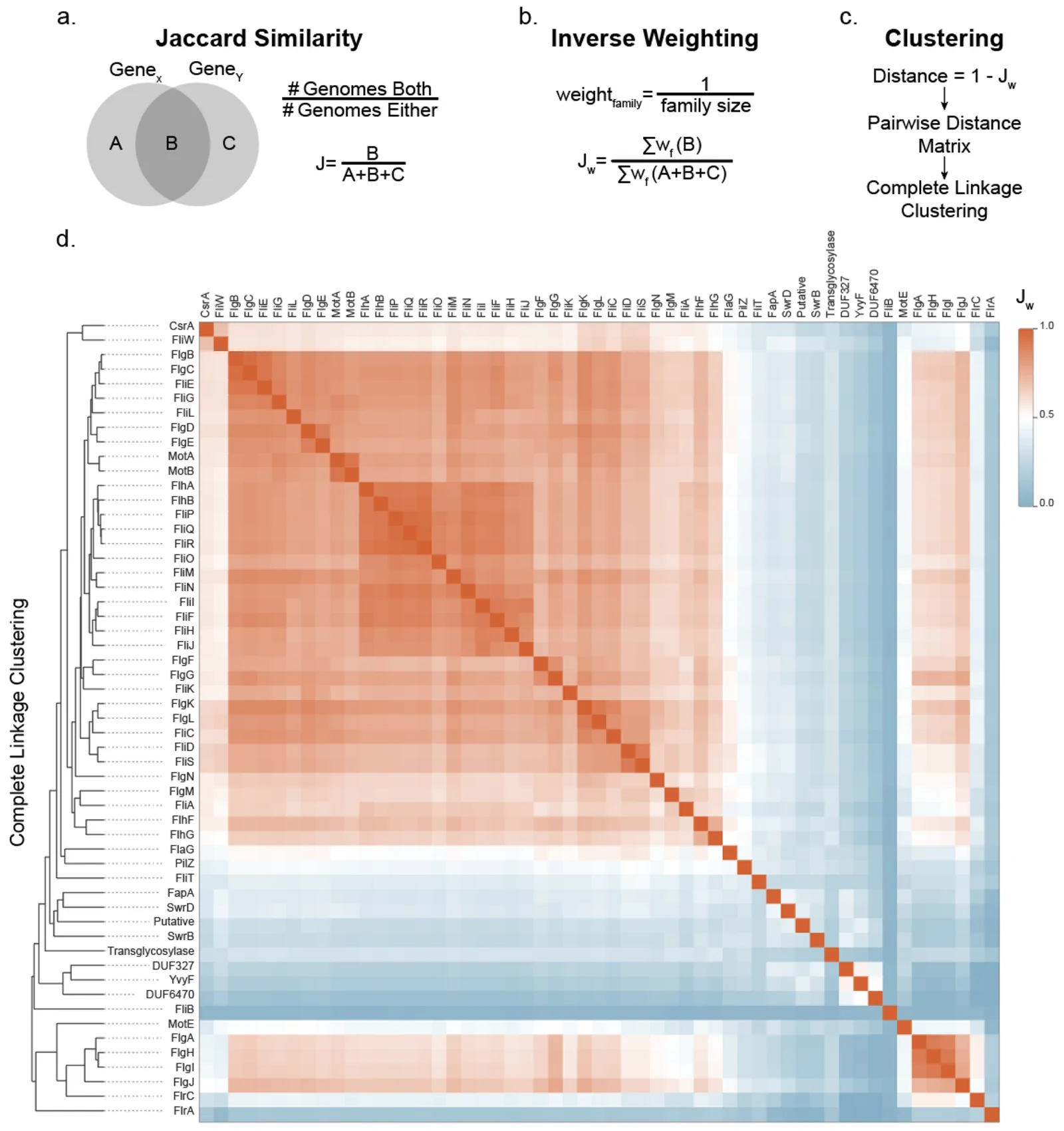
Pairwise co-presence analysis identifies conserved and auxiliary flagellar gene clusters. **a,** Schematic representation of the Jaccard similarity index, calculated as the fraction of genomes in which two genes co-present relative to the number of genomes containing at least one of the two genes. **b,** To correct for uneven taxonomic sampling, each genome was weighted inversely to the number of representative genomes in its family, yielding a family-balanced weighted Jaccard similarity in which all families contributed equally. **c,** Weighted Jaccard similarities were converted to distances (1 – J_w_) and used for hierarchical clustering with complete linkage. **d,** Heatmap of pairwise family-weighted Jaccard similarities for 55 widely distributed flagellar genes across GTDB representative bacterial proteomes (release 214). Genes are ordered by complete-linkage hierarchical clustering, shown as a dendrogram on the left. The heatmap resolves broadly conserved core flagellar genes as well as more weakly associated auxiliary components.

Following the identification of flagellar homologs, we quantified pairwise gene co-presence across bacterial genomes to identify flagellar genes that are preferentially retained together. The rationale was that genes encoding components of the same functional module, and especially those required for a functional flagellum, should exhibit correlated presence across genomes. We therefore analyzed 53 abundant flagellar genes and quantified pairwise co-presence using the Jaccard similarity index, defined as the fraction of genomes containing both genes among those containing at least one of the two (Fig. 2a). To account for taxonomic sampling bias, each genome was weighted inversely to the number of representative genomes in its taxonomic family, yielding a balanced weighted Jaccard similarity (J_w_) in which all families contributed equally (Fig. 2b). Weighted similarities were converted to distances (1 – J_w_) and used for complete-linkage clustering (Fig. 2c).

This analysis resolved two major clusters of highly co-present genes (J_w_ generally > 0.5). The larger cluster comprised 36 genes ranging from CsrA to FlhG on Fig. 2, whereas the smaller cluster contained four genes from FlgA to FlgJ. Although separated by hierarchical clustering, these groups also showed strong cross-cluster similarity, indicating that they are frequently retained together across bacterial genomes. The smaller cluster likely separates because it contains proteins associated with the outer membrane, whose presence depends on monoderm versus diderm cell-envelope architecture. Together, these two clusters define a set of 40 strongly co-present flagellar genes, consistent with a conserved functional core and highlighting them as candidate components of the ancestral flagellar gene set.

Beyond identifying a broadly conserved core, co-presence analysis also recovered local clusters that recapitulate known functional organization within the flagellum. These finer-scale associations provide an unsupervised view of flagellar architecture, in which genes involved in the same substructure, assembly step, or regulatory pathway tend to cluster together. The most tightly correlated sub-cluster comprised 12 type-III secretion system (T3SS) genes (FliJ, FliI, FliF, FliH, FliN, FliM, FliO, FlhA, FlhB, FliQ, FliP, and FliR), likely reflecting their shared occurrence in both flagellar and non-flagellar T3SS, which were not explicitly excluded by our search pipeline. Additional coherent clusters corresponded to well-defined structural modules, including the stator unit (MotA, MotB), proximal rod components (FlgB, FlgC, FliE), distal rod components (FlgF, FlgG), P and L rings (FlgA, FlgH, FlgI, FlgJ), hook (FlgD, FlgE), hook–filament junction and filament (FlgK, FlgL, FliC), filament cap and chaperone of the filament (FliD, FliS). Regulatory proteins also clustered according to known functional relationships, including FliA/FlgM, FlhF/FlhG, and CsrA/FliW.

Beyond recovering known functional associations, these local clusters reveal potential links among poorly characterized flagellar components, including associations between YvyF and DUF327/DUF6470 proteins, MotE and P- and L-ring proteins, SwrD and FapA, PilZ-domain proteins and FlaG, and FlaF and FlbT (Fig. 2d; Supplementary Fig. 1). These associations provide testable hypotheses for future functional studies.

### Phyletic distribution supports 52 ancestral flagellar components

**Figure 3:**
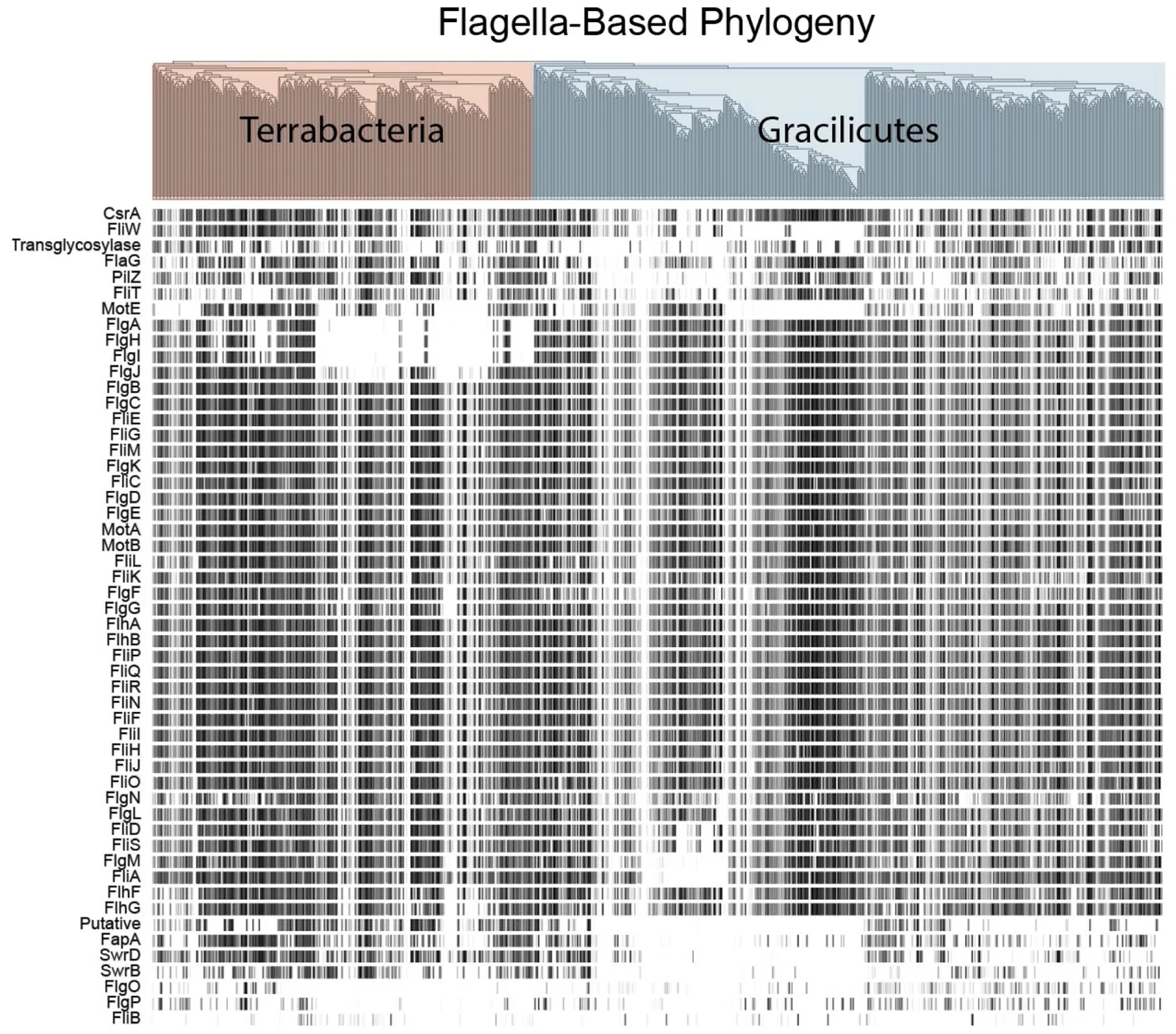
Phyletic distribution of flagellar proteins supports the ancestral flagellar gene set. Presence/absence patterns for the 52 inferred ancestral flagellar genes are shown across the taxonomic order-level flagella-based phylogeny displayed at the top. The phylogeny separates two major clades, corresponding primarily to Terrabacteria (red), and Gracilicutes (blue). Each vertical column represents a bacterial taxonomic order, and black marks indicate the presence of a given gene within genomes belonging to that order. Orders were normalized to equal horizontal width regardless of the number of sampled genomes, allowing patterns of gene presence and absence to be compared independently of uneven taxonomic sampling.

Although 40 flagellar genes showed strong co-presence across bacterial genomes, co-presence alone does not establish ancestral presence. To support ancestry, a gene must also exhibit broad distribution across deeply diverged bacterial lineages in a pattern more consistent with vertical inheritance than with recent horizontal transfer. To assess this, we reconstructed a flagella-based phylogeny at the taxonomic order level by integrating phylogenetic signals across 74 individual flagellar gene trees (Fig. 3). By averaging relationships at the order level, this approach was designed to recover the dominant vertical inheritance signal shared across flagellar components while reducing the noise introduced by horizontal gene transfer, lineage-specific gene loss, recent acquisitions, and divergence within lower-level lineages.

For each gene tree, leaves were replaced with their corresponding taxonomic orders, and shared subtree membership among orders was quantified to generate an order-level similarity matrix. These matrices were averaged across all 74 flagellar gene trees and converted into a distance matrix, from which we inferred a neighbor-joining tree summarizing the shared evolutionary signal across flagellar proteins. The resulting topology resolved two major superclades corresponding predominantly to Gracilicutes and Terrabacteria (Fig. 3), providing a natural framework for inferring ancestral gene presence and for placing the root of the tree between these two clades.

We reasoned that genes broadly distributed across both superclades, in the absence of evidence for recent horizontal transfer, were most consistent with presence in the LBCA.

Mapping gene presence onto this phylogeny supported 52 ancestral flagellar proteins, more than doubling previous estimates of the ancestral flagellar gene set. Most were broadly distributed and highly co-present, consistent with long-term vertical inheritance and functional conservation. However, several inferred ancestral genes were not highly abundant in extant genomes, suggesting frequent secondary loss. This pattern was especially pronounced for *flgO*, *flgP*, and *fliB*. Among these, *flgO* and *flgP* remain strong candidates for ancestral components despite their lower abundance. Both genes are enriched in diderm bacteria across both major superclades and frequently co-occur with the L- and P-ring genes (*flgA*, *flgH*, and *flgI*), consistent with their known roles in outer membrane-associated flagellar structures^24,25^. Their patchier distribution is therefore more parsimoniously explained by repeated loss in monoderm lineages than by recent acquisition. By contrast, the evidence for ancestral presence is weaker for *fliB* due to its sporadic distribution within both superclades and is therefore less easily distinguished from lineage-specific gain or horizontal transfer. In addition to ancestral components, phyletic mapping also identified lineage-restricted innovations, including *yvyF*, *DUF327*, and *DUF6470* in the common ancestor of Gracilicutes, and *flrA* and *flrC* in the common ancestor of Terrabacteria. The remaining 22 flagellum-associated genes showed restricted distributions, consistent with auxiliary components acquired later in flagellar evolution.

### Gene Retention and Absence for Ancestral Core Components

**Figure 4:**
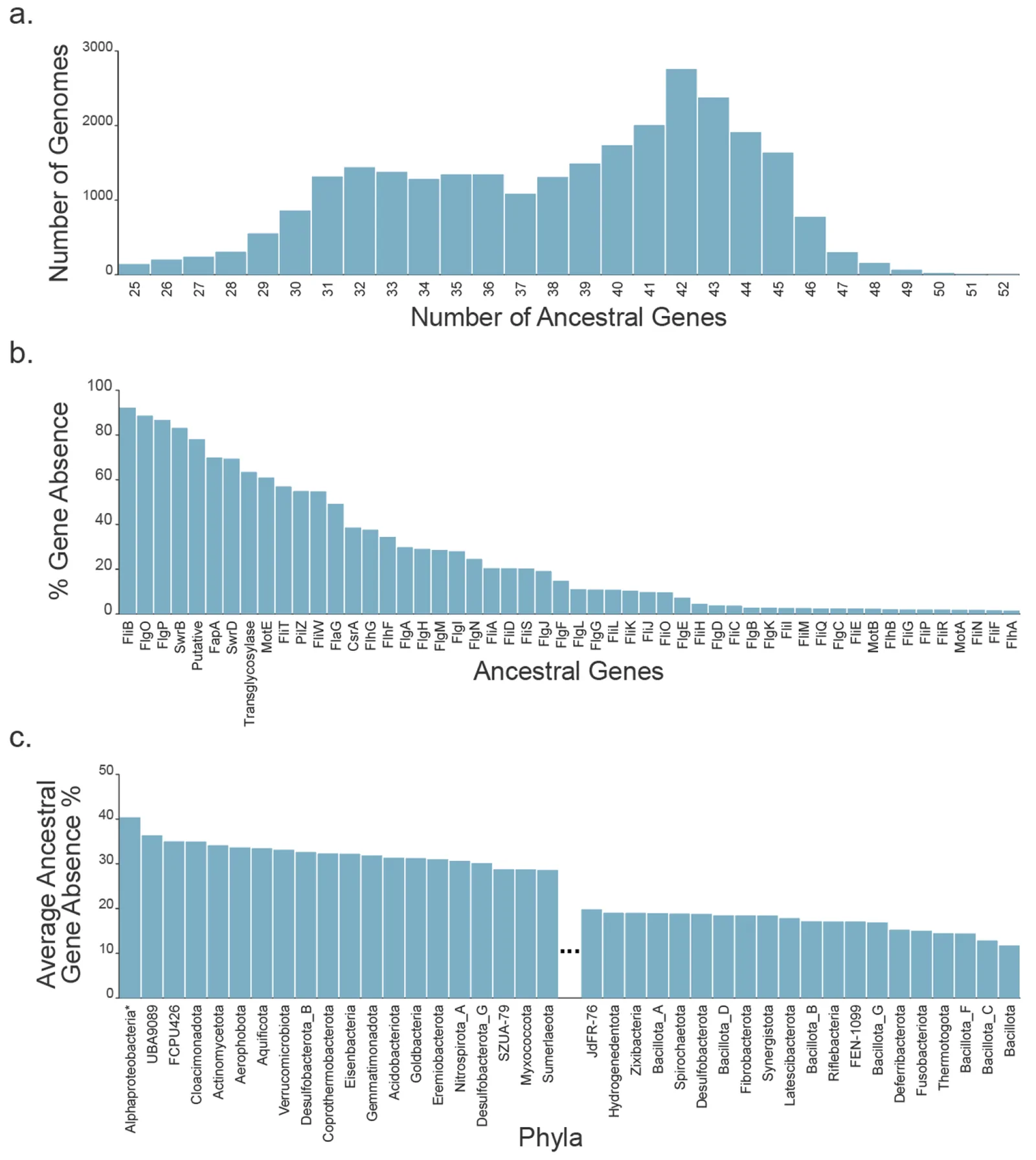
Retention and absence patterns of ancestral flagellar proteins. **a,** Distribution of retained ancestral flagellar proteins across genomes encoding at least 25 of the 52 inferred ancestral genes. **b,** Gene-specific absence frequencies for each ancestral flagellar protein across the same genome set, shown as the percentage of genomes in which a given gene is absent. **c,** Total ancestral gene absence percentages across bacterial phyla. For visualization, only the top 20 and bottom 20 phyla (restricted to phyla with at least 5 members) are shown. To minimize biases associated with differences in cell-envelope architecture, outer membrane-associated proteins (*FlgA*, *FlgH*, *FlgI*, and *FlgJ*) were excluded from this analysis. Because of its large size and phylogenetic diversity, *Pseudomonadota* are shown at the class level.

We next examined retention and absence patterns among the 52 inferred ancestral flagellar proteins. Across genomes encoding at least 25 core components, most retained between 41 and 44 ancestral proteins (Fig. 4a), consistent with the observation that many extant flagellated bacteria encode more than 40 flagellar components. However, no extant genome retained the complete inferred ancestral set. Notably, genomes encoding at least 49 ancestral components were found in both Gracilicutes and Terrabacteria, indicating that highly complex flagellar systems are not restricted to a single bacterial lineage and supporting the presence of a similarly complex apparatus before the divergence of these two major superclades.

We next quantified absence frequencies for individual ancestral genes (Fig. 4b). Here, gene absence refers to lack of detection in our dataset and does not necessarily indicate true evolutionary gene loss, as some genes may be missing because of incomplete genomes, sequence divergence, or limitations of our pipeline. Genes encoding core structural, export, and motility functions, including *flhA*, *flhB*, *motA*, *motB, fliQ*, *fliR*, *fliP*, *fliC, fliG*, and *fliE*, were absent from only ~2–5% of genomes. By contrast, regulatory and accessory genes showed substantially higher absence frequencies. The retention pattern is consistent with differences in functional constraints: genes required for assembly, export, and motility are strongly retained due to their indispensable roles. In contrast, genes with regulatory or accessory functions are more frequently absent, perhaps reflecting lineage-specific selective pressures. The absence of any extant genome containing all 52 ancestral proteins is therefore best explained by repeated lineage-specific loss of less constrained components.

To assess broader phylogenetic trends, we next quantified total gene absence percentages at the phylum level (Fig. 4c). Because *Pseudomonadota* is both highly represented and phylogenetically diverse, its classes were analyzed separately. To avoid bias associated with cell-envelope architecture, outer membrane-associated proteins (*FlgA*, *FlgH*, *FlgI*, and *FlgJ*) were excluded from this comparison, and lineages represented by fewer than five genomes were omitted. The lowest average absence percentages (~15%) were observed in several Bacillota-associated lineages (*Bacillota*, *Bacillota_C*, *Bacillota_F*, and *Bacillota_G*), as well as *Fusobacteriota*, *Thermotogota*, *Riflebacterota*, and *Deferribacterota*. By contrast, *Alphaproteobacteria* showed the highest average loss (>40%), followed by *Cloacimonadota*, *Actinomycetota*, and the candidate phyla *UBA9089* and *FCPU426*, each with loss rates near 35%. The lineage-specific differences indicate that, although most ancestral components are broadly retained, the extent of secondary loss varies substantially across bacterial groups.

Together, these results support a model in which the bacterial flagellum was already a highly complex system in the last bacterial common ancestor, but extant flagellar repertoires have been shaped by differential retention and repeated secondary loss. Structural, export, and motility proteins form a strongly retained functional core, whereas regulatory and accessory components have been more frequently lost.

## DISCUSSION

**Figure 5.**
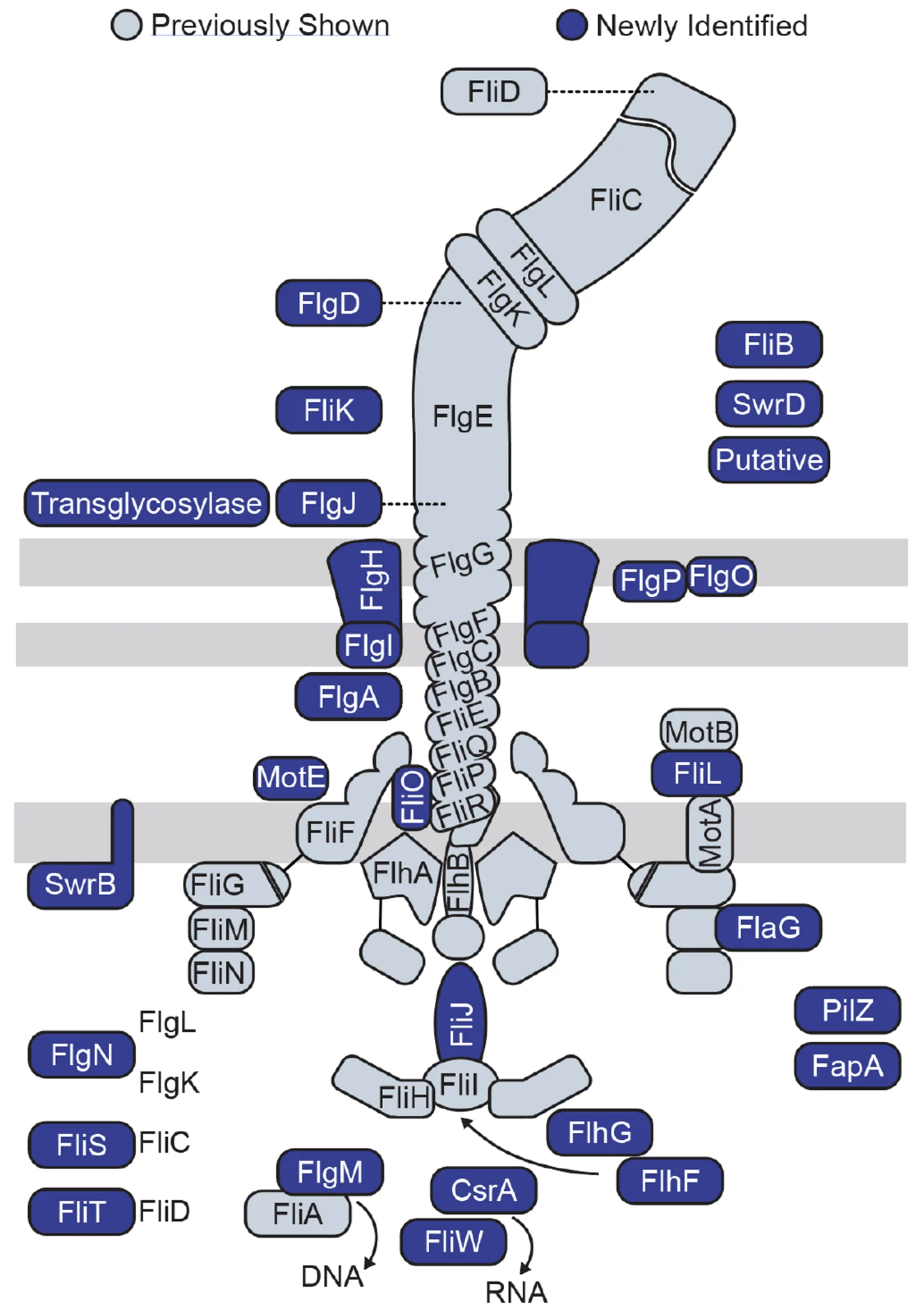
Reconstruction of the ancestral bacterial flagellum. Schematic overview of the flagellar system inferred for the LBCA based on phyletic distribution, co-presence analysis, and large-scale homology searches. Proteins previously recognized as ancestral components are shown in gray, whereas additional ancestral proteins identified in this study are shown in dark blue. Together, these components define an ancestral flagellum substantially more complex than previous reconstructions. An extended version incorporating major lineage-specific flagellum-associated proteins is provided in Supplementary Figure 2.

The bacterial flagellum has long served as a model for the evolution of complex molecular systems ^13,14,26,27^, yet its evolutionary history has remained unresolved. Previous reconstructions proposed that the last bacterial common ancestor encoded 24 ancestral flagellar genes ^14^, implying that modern flagella might have been assembled gradually through sequential addition of new components. Our results support a different scenario. By combining sensitive large-scale homolog detection with co-presence analysis, phyletic mapping, and presence/absence profiling, we infer the presence of 52 flagellum-associated proteins in the LBCA (Fig. 5), thus more than doubling previous estimates.

This reconstruction supports a “complexity-first” model of flagellar evolution from the LBCA onward. Instead of emerging as a minimal scaffold that was progressively elaborated within Bacteria, the bacterial flagellum appears to have already existed in the LBCA as a complex system whose descendants were later modified by differential retention, loss, and lineage-specific innovation. This view is consistent with the “big bang” model, in which major evolutionary transitions are marked by the early emergence of comparatively complex biological systems, followed by a slower subsequent period of refinement ^1,28^. Therefore, increased flagellar complexity may have provided early bacterial lineages with an important ecological advantage, enhancing their ability to explore diverse environments and contributing to the long-term persistence of flagellated descendants. Subsequent flagellar evolution appears to have been dominated primarily by secondary loss and lineage-specific innovation. During this slower phase, most extant flagellated bacteria retained over 40 flagellar components (Fig 4a), indicating that much of the ancestral complexity was preserved. However, gene retention patterns show that this preservation was not uniform. Structural, export-associated, and motility-associated components are retained at much higher frequencies (Fig. 4b), consistent with strong selective pressure to preserve the essential architecture and function of the flagellum. In contrast, regulatory and accessory components are lost more frequently, likely because their functions are more readily compensated or rewired in different cellular and ecological contexts. Lastly, differences in gene absence patterns across lineages (Fig. 4c) suggest that this slower phase did not proceed uniformly but instead involved lineage-specific trajectories of flagellar reduction and remodeling.

The co-presence analysis (Fig. 2) further shows that this ancestral complexity was modular ^29^. In addition to defining a broadly conserved 40-gene core, pairwise co-presence recaptured known functional modules, including the export apparatus, stator, rod, hook, filament, and several regulatory circuits (Supplementary Figure 1). Each core module remains recognizable across deep bacterial divergence, indicating that much of the flagellum’s functional organization was established early and subsequently retained. This suggests that the ancestral flagellum was not only complex, but already organized into semi-independent functional units that could be differentially modified ^30^.

Our findings also highlight a broader methodological point. Deep evolutionary reconstructions are often limited by the difficulty of detecting highly diverged homologs and distinguishing true gene absence from search failure. To improve sensitivity, we performed sequence searches using relaxed E-value thresholds and primarily relied on phylogenetic clustering homologous sequences from noise. Despite these efforts, we identified cases in which previously characterized homologs showed unexpectedly low scores against the HMM profiles or iterative sequence profiles used in our initial searches. For FliF, FliH, FlrC and MotB, we addressed this by rerunning the pipeline using alternative homologs from lineages where the initial search failed to recover clear candidates. These examples demonstrate that even well-constructed automated searches require targeted inspection in certain cases to distinguish true gene loss from failure to detect highly diverged homologs. Similarly, very short or unusually long protein sequences may occasionally cluster within retrieved homologous clades during high-throughput analysis. Therefore, although the retention and absence patterns reported here provide a broad evolutionary framework, they remain the product of a large-scale automated pipeline and could be further refined through additional targeted curation. To support further refinement through community feedback, we provide the dataset generated in this study through flagelladb.org, where researchers can easily access the results and explore flagellar gene evolution across bacterial lineages.

Taken together, our results support a revised view of flagellar evolution. The LBCA likely possessed a structurally and functionally sophisticated flagellum similar to those in many modern bacteria. Subsequent evolution was driven primarily by selective retention, repeated loss, and lineage-specific remodeling of an already complex ancestral machine, whereas the evolutionary steps that gave rise to this system remain to be resolved.

## METHODS

### Homology searches and sequence retrieval

For each flagellar gene, we used HMMs from the KofamKOALA^22^ and Pfam^23^ databases when available. When no HMMs were available, we used previously annotated protein sequences as search queries. HMM searches were performed with HMMER v3.3.2^31^ using the “-E 1000” option. Iterative profile searches using protein sequences were performed with MMSeqs2^32^ using the “-e 1000 --e-profile 10 -s 7.5” options. All database searches were conducted against GTDB release 214^33^ proteomes, covering 80,789 representative bacterial genomes.

For most genes, we retrieved the top 50,000 hits from each search. This cutoff was chosen empirically because our initial analyses suggested that nearly one third of genomes in GTDB r214 contain flagellar genes, and we therefore aimed to exceed the expected number of true homologs to maximize sensitivity. For multicopy proteins such as FliC, MotA, and MotB, we retrieved the top 100,000 hits using the same reason.

### Filtering of Inferred Homologs

#### Sequence Alignment and Gene Tree Inference

For each set of retrieved sequences, we generated a multiple sequence alignment (MSA) using FAMSA v2.2.2^34^ with default settings. Alignment columns containing more than 90% gaps were trimmed using trimAl v1.4^35^ with the “-gt 0.1” option, and gene trees were inferred from the trimmed alignments using FastTree v2.1.11^36^. For searches based on HMM models, we performed this workflow both on the full retrieved sequences and on the regions matched by the HMM model. For each flagellar component, we then manually selected the tree that provided the clearest separation of homologous sequences.

#### Ordering the Gene Tree and MSA

Next, tree leaves were ordered by HMM or bit scores using a recursive algorithm. First, each tree was rooted using the lowest-scoring sequences as an outgroup. We then traversed the tree from the root through all internal nodes and, for each pair of sister clades, calculated the average HMM or bit score. When necessary, sister clades were rearranged so that the clade with the higher average score appeared on the left. This ensured that high-scoring sequences were concentrated on the left side of the tree. The resulting tree order was also applied to the corresponding MSAs.

We then plotted HMM or bit scores together with sequence rank in the search results to identify tree regions enriched in high-scoring sequences that also ranked highly in the original search output.

#### Identifying Gene-Gene Neighbors

We then compared the sequences in each gene search output against up to 100,000 sequences from the outputs of the other flagellar gene searches. For each sequence, we identified genes located on the same genome, contig, and strand, and classified a gene as a neighbor if it was located within 500 bp of the query sequence. We then aggregated this neighborhood information into 50 windows along the ordered tree and plotted it as a line plot to highlight tree regions associated with specific neighboring genes.

#### Identification Criteria

To distinguish true homologs from noise, we evaluated four main criteria. True homologous sequences were expected to cluster within clades because of their shared ancestry and to exhibit higher HMM or bit scores because of stronger similarity to the query model or protein sequence. We also assessed whether these sequences were frequently located near other flagellar genes and displayed visually distinct patterns in the MSA. Sequences within clade(s) that satisfied these criteria were selected for downstream analyses. Overall, this approach integrates phylogenetic clustering, sequence similarity, gene neighborhood context, and MSA patterns to identify homologous sequences across bacteria. All the MSAs, trees database searches and plots are available at our Zenodo repository.

### Gene Co-presence Analysis

#### Measuring Gene Co-presence

For each pair of flagellar genes, we quantified their co-presence across bacterial genomes using the Jaccard similarity index. For a given gene pair, Jaccard similarity index is calculated as number of genomes in which both genes were detected divided by number of genomes in which at least one of the two genes was detected (Fig. 2a).

To reduce the influence of uneven taxonomic sampling, we applied family-level inverse weighting. Each genome was inversely weighted according to the number of genomes sampled from its bacterial family, such that genomes from highly represented families contributed less individually than genomes from sparsely represented families. For each gene pair, the weighted contributions of genomes containing both genes were summed and divided by the summed weighted contributions of genomes containing at least one of the two genes (Fig. 2b). This produced a family-balanced weighted Jaccard similarity index (J_w_) for each gene pair.

#### Hierarchical Clustering

The resulting pairwise similarity scores were converted to distances by subtracting weighted Jaccard indexes from one (Fig. 2c). Genes were then grouped using complete-linkage hierarchical clustering, and the clustered similarity matrix was visualized as a heatmap (Fig. 2d).

### Constructing Flagella-based Phylogeny

#### Selection of flagellar gene trees

To construct a flagella-based phylogeny, we summarized the phylogenetic signal contained across individual flagellar gene trees rather than concatenating sequences into a single alignment. First, we extracted the inferred homologs for each flagellar gene from the manually selected clades described above. For this analysis, trees generated from full protein sequences and HMM-detected regions together. As a result, we analyzed 74 gene trees associated with 37 flagellar genes.

These genes included FlgB, FlgC, FlgD, FlgE, FlgK, FlgL, FlhA, FlhB, FliC, FliD, FliE, FliF, FliG, FliH, FliI, FliK, FliM, FliN, FliP, FliQ, FliR, FliS, MotA, MotB, FliO, FliJ, FliL, FliW, FlaG, FlgM, FlgN, FlhG, FlhF, FlgJ, FlgG, FlgF, and Transglycosylase. These genes were selected because they are broadly distributed across diverse bacterial lineages, allowing us to maximize phylogenetic coverage while keeping the number of analyzed trees manageable.

#### Order-level labeling of gene trees

For each gene tree, all leaf nodes were labeled with their corresponding GTDB r214 order-level taxonomic assignment based on the genome from which each sequence originated. Orders represented by fewer than two sequences in a given tree were excluded from that tree to reduce the influence of poorly sampled lineages. We then traversed the hierarchy of each gene tree from the root toward the terminal leaves. At each internal node, the algorithm identified all descendant leaves and counted how many sequences belonged to each order. Internal nodes containing two or more orders contributed to an order-by-order similarity matrix.

#### Normalization of order representation within each tree

For each internal node, the sequence count for each order was scaled by dividing it by the total number of sequences from that order in the full gene tree raised to 0.8. This partial inverse weighting prevented highly represented orders from dominating the similarity matrix without fully equalizing them with sparsely represented orders. At the same time, the scaling preserved stronger associations supported by a greater number of descendant leaves, reducing the influence of horizontal gene transfer events.

#### Calculation of order-pair similarity

For a given order pair at a given node, their co-representation was calculated as the product of their scaled descendant counts under that node. This calculation was performed for every pair of orders present under a given node, and the resulting values were added to the corresponding entries in the order-by-order similarity matrix.

As this procedure was repeated across all internal nodes in the tree, order pairs accumulated higher similarity values when their homologs repeatedly occurred together within shared internal clades. After all nodes were evaluated, the resulting matrix represented the order-level phylogenetic similarity encoded by that individual gene tree.

#### Combining similarity matrices across gene trees

Each gene-tree similarity matrix was then normalized independently so that all trees contributed equally to the final analysis. The normalized matrices from all 74 trees were then combined into a single order-level similarity matrix summarizing the cumulative phylogenetic signal across the analyzed flagellar gene trees. The final similarity matrix was restricted to orders that were included in at least 80% of the trees analyzed.

#### Construction of the distance matrix and neighbor-joining tree

Finally, the combined order-level similarity matrix was converted into a distance matrix by subtracting each similarity value from one. In this distance matrix, order pairs with high co-representation across flagellar gene trees had shorter distances, whereas order pairs with low co-representation had longer distances. The resulting distance matrix was then used to construct a neighbor-joining tree. This tree represents a flagella-based phylogeny that summarizes the collective phylogenetic signal shared across the analyzed flagellar genes.

### Calculating Gene Retention and Absence Percentages

#### Calculating ancestral gene retention per genome

To evaluate the retention of ancestral flagellar components across bacteria, To ensure the analysis focused on lineages with substantial flagellar representation, the dataset was filtered to include only genomes encoding at least 25 of these 52 ancestral components. For each genome in this filtered dataset, the total number of present ancestral genes was plotted as a bar plot in Figure 4a. Here, retention is defined as the detection of at least one homolog for a given gene.

#### Calculating gene-specific absence percentages

To assess the evolutionary constraints on individual flagellar components, we calculated the absence frequency for each of the 52 ancestral genes. Using the same filtered dataset of genomes containing 25 or more ancestral genes, we calculated percent gene absence as the number of genomes in which each specific gene is present divided by the total number of genomes. The genes were then ranked in descending order based on their absence percentages to identify components most and least prone to secondary evolutionary loss (Fig. 4b).

#### Calculating lineage-specific gene absence

To measure rate of gene absence across broader taxonomic groups, we calculated average gene absence percentages at the phylum level. To prevent biases originating from fundamental differences in cell-envelope architecture, outer membrane-associated genes (*flgA*, *flgH*, *flgI*, and *flgJ*) were excluded from this calculation, leaving a subset of 49 ancestral genes. Furthermore, because of its large size and broad phylogenetic diversity, the phylum *Pseudomonadota* was analyzed at the class level. Lineages represented by fewer than five genomes were excluded to minimize sampling artifacts. For each analyzed lineage, the gene-specific absence percentages were summed and divided by 49 to calculate the average ancestral gene loss percentage. Finally, lineages were sorted by this average absence metric, and the top 20 and bottom 20 lineages were visualized for comparison (Fig. 4c).

## Supplementary Material

Supplement 1

Supplement 2

## Figures and Tables

**Table 1.** Orthologous groups of flagellar components with different names.

Group ID	Protein Name	Organism	Accession	Reference
FliN	FliN	*S. enterica*	NP_460930.1	^37^
	FliY	*B. subtilis*	NP_389514.1	^38^
MotA	MotA	*B. subtilis*	NP_389252.1	^39^
	MotP	*B. subtilis*	NP_390851.1	^40^
	PomA	*V. cholerae*	WP_001922974.1	^41^
MotB	MotB	*B. subtilis*	NP_389251.1	^39^
	MotS	*B. subtilis*	NP_390850.1	^40^
	PomB	*V. cholerae*	WP_000340821.1	^41^
FliC	FliC	*S. enterica*	NP_460912.1	^42^
	FlaA	*S. putrefaciens*	WP_011919615.1	^43^
	FlaB	*S. putrefaciens*	WP_011919614.1	^43^
	Hag	*B. subtilis*	NP_391416.1	NA
	FlaA	*C. jejuni*	YP_002344727.1	^44^
	FlaB	*C. jejuni*	YP_002344726.1	^44^
MotE	MotE	*S. meliloti*	WP_002721883.1	^45^
	FlgY	*C. jejuni*	YP_002344876.1	^46^
	FlbB	*B. burgdorferi*	WP_014023531.1	^47^
	YlxF	*B. subtilis*	NP_389508.2	NA
SwrB	SwrB	*B. subtilis*	NP_389530.1	^48^
	FlgW	*C. jejuni*	YP_002343622.1	^49^
YdiV	YdiV	*S. enterica*	NP_460310.1	^50^
	YhjH	*E. coli*	NP_417982.2	^51^
YcgR	YcgR	*E. coli*	NP_415712.1	^52^
	PlzD	*V. cholerae*	WP_017422363.1	^53^
	MotI/DgrA	*B. subtilis*	NP_390172.1	^54^
	FlgZ	*P. aeruginosa*	WP_003119635.1	^55^
MotL	MotL	*S. putrefaciens*	WP_077750673.1	^56^
	MapZ	*P. aeruginosa*	WP_003094864.1	^57^
	PlzA	*V. cholerae*	WP_001893327.1	^53^
FlrA/FlaK	FlrA	*V. cholerae*	WP_001190986.1	^58^
	FlbD	*C. crescentus*	YP_002516327.1	^59^
	FlgR	*C. jejuni*	YP_002344419.1	^60^
FlgF	FlgF	*S. enterica*	NP_460149.1	^61^
	FlhO	*B. subtilis*	NP_391521.1	^62^
FlgG	FlgG	*S. enterica*	NP_460150.1	^61^
	FlgP	*B. subtilis*	NP_391520.2	^62^

**Table 2.** Proteins involved in assembly, function, and regulation of bacterial flagella.

Protein or orthologous group	Function	Organism	Accession	Reference
FliE	Adaptor at MS-ring/rod junction	*S. enterica*	NP_460921.1	^63,64^
FliF	MS-ring protein	*S. enterica*	NP_460922.1	^65^
FliG	Rotor torque generator, C-ring	*S. enterica*	NP_460923.1	^65,66^
FliH	Peripheral stalk/regulator of FliI ATPase	*S. enterica*	NP_460924.1	^67,68^
FliI	Protein export ATPase	*S. enterica*	NP_460925.1	^67^
FliJ	Export apparatus stalk / chaperone–ATPase adaptor	*S. enterica*	NP_460926.1	^67^
FliK	Hook-length control protein	*S. enterica*	NP_460927.1	^69^
FliL	Stator-associated periplasmic protein	*S. enterica*	NP_460928.1	^70^
FliM	C-ring subunit	*S. enterica*	NP_460929.1	^71^
FliN	C-ring small subunit	*S. enterica*	NP_460930.1	^71^
FliO	Assembly factor for FliP assembly into the export gate	*S. enterica*	NP_460931.3	^72^
FliP	Core export-gate channel subunit	*S. enterica*	NP_460932.1	^73^
FliQ	Export-gate subunit	*S. enterica*	NP_460933.1	^73^
FliR	Export-gate subunit	*S. enterica*	NP_460934.1	^73^
FlhA	Core export-gate channel	*S. enterica*	NP_460870.1	^68^
FlhB	Export gate switch protein	*S. enterica*	NP_460871.1	^73^
FlhE	periplasmic protein associated with export/motor function	*S. enterica*	NP_460869.1	^74^
FlhF	flagellar placement and number regulator (SRP-type GTPase)	*V. alginolyticus*	WP_226421005.1	^75^
FlhG	MinD-like ATPase limiting flagellar number	*B. subtilis*	WP_010886507.1	^76^
FlgA	Periplasmic P-ring assembly chaperone	*S. enterica*	NP_460144.1	^77^
FlgB	Proximal rod subunit	*S. enterica*	NP_460145.1	^61^
FlgC	Rod subunit (inner/middle)	*S. enterica*	NP_460146.1	^61^
FlgD	Hook cap protein	*S. enterica*	NP_460147.1	^78^
FlgE	Hook subunit	*S. enterica*	NP_460148.1	^61^
FlgF	Proximal/distal rod protein	*S. enterica*	NP_460149.1	^61^
FlgG	Distal rod subunit	*S. enterica*	NP_460150.1	^61^
FlgH	L-ring subunit	*S. enterica*	NP_460151.1	^79^
FlgI	P-ring subunit	*S. enterica*	NP_460152.1	^79^
FlgJ	Rod assembly protein with muramidase activity	*S. enterica*	NP_460152.1	^80^
FlgK	Hook–filament junction protein 1	*S. enterica*	NP_460154.1	^81^
FlgL	Hook–filament junction protein 2	*S. enterica*	NP_460155.1	^81^
FlgO	Outer membrane ring protein	*V. alginolyticus*	WP_005382013.1	^82^
FlgT	T-ring protein of the polar flagellar motor	*V. alginolyticus*	WP_048626114.1	^82^
FlgP	Polar flagellar outer ring (H-ring) protein	*C. jejuni*	YP_002344421.1	^83^
FlgQ	Rod/P-ring assembly factor	*C. jejuni*	YP_002344420.1	^83^
MotX	Accessory stator-associated protein; TPR protein, required for stator formation in Na-driven motor	*V. alginolyticus*	WP_005387944.1	^84,85^
MotY	T-ring stator assembly protein, required for stator formation in Na-driven motor	*V. alginolyticus*	WP_012841571.1	^85,86^
FliC	Flagellin (major filament protein)	*S. enterica*	NP_460912.1	^42^
FliD	Filament cap protein	*S. enterica*	NP_460913.1	^87^
FliS	Flagellin-specific export chaperone	*S. enterica*	NP_460914.1	^88^
FliT	Chaperone for FliD	*S. enterica*	NP_460915.1	^88^
MotA	Stator A subunit	*S. enterica*	NP_460880.1	^89^
MotB	Stator B subunit	*S. enterica*	NP_460879.1	^89^
FlgM	Anti-σ^28^ factor controlling late flagellar gene expression	*S. enterica*	NP_460143.1	^90^
FlgN	FlgK/FlgL chaperone	*S. enterica*	NP_460142.1	^91^
FliZ	Post-transcriptional / transcriptional regulator	*S. enterica*	NP_460908.1	^92^
FlrA	σ^54^-dependent enhancer-binding protein	*V. cholerae*	WP_001190986.1	^58^
FlrC	σ^54^-dependent enhancer-binding protein	*V. cholerae*	WP_000079330.1	^93^
FlhC	Flagellar transcriptional activator	*E. coli*	NP_416405.1	^94^
FlhD	Flagellar transcriptional activator	*E. coli*	NP_416406.4	^94^
FliA	σ^28^ flagellar sigma factor	*S. enterica*	NP_460909.1	^95^
YdiV	anti-FlhD_4_C_2_ / FlhDC regulator	*S. enterica*	NP_460310.1	^50^
YcgR	c-di-GMP–dependent flagellar motor brake protein	*E. coli, S. enterica*	NP_415712.1, NP_460754.1	^52,96^
MotL	c-di-GMP –dependent flagellar motor regulator	*S. putrefaciens*	WP_077750673.1	^56^
FlgX	chaperone for MotAB stator units	*C. jejuni*	YP_002343623.1	^97^
CsrA	RNA-binding global regulator	*E. coli*	NP_417176.1	^98^
FlaG	Modulator of filament assembly	*V. cholerae*	WP_000405330.1	^99^
FliW	CsrA antagonist	*B. subtilis*	NP_391418.1	^100^
MotE	auxiliary flagellar motor protein	*S. meliloti*	WP_002721883.1	^45^
MotC	periplasmic motility protein	*S. meliloti*	WP_014529017.1	^45^
PflA	Distal spoke-ring protein	*H. pylori*	NP_208066.1	^101^
PflB	Distal spoke-ring protein	*H. pylori*	NP_208270.1	^101^
MotK	Periplasmic protein	*C. sphaeroides*	WP_002721885.1	^102^
SwrA	Swarming/flagellar gene expression control	*B. subtilis*	WP_015251121.1	^103^
SwrB	flagellar basal body activator	*B. subtilis*	NP_389530.1	^48^
SwrD	Torque enhancer	*B. subtilis*	YP_003097728.1	^104^
FliB	Flagellin-specific lysine-N-methylase	*S. enterica*	NP_460911.1	^105^
FljA	Repressor of FliC expression	*S. enterica*	NP_461697.1	^106^
FlcA	Flagellar collar protein, TPR	*B. burgdorferi*	NP_212460.1	^47^
FlcB	Flagellar collar protein	*B. burgdorferi*	NP_212192.1	^47^
FlcC	Flagellar collar protein	*B. burgdorferi*	NP_212758.1	^47^
FlcD	Flagellar collar protein	*B. burgdorferi*	NP_212370.1	^47^
FlaY	FliD analog	*C. crescentus*	NP_420277.1	^107^
FlaF	flagellin chaperone, FliS analog	*C. crescentus*	NP_420272.2	
PleA	Lytic transglycosylase	*C. crescentus*	WP_012640469.1	^108^
FlbT	Posttranslational regulator of flagellin	*C. crescentus*	NP_420271.1	^109^
FapA (DUF342)	Regulator of flagellar biosynthesis and motility	*V. vulnificus*	WP_015728486.1	^110^
Flagellar_put (Putative)	Function unknown	*A. longum*	WP_004096850.1	This study
YvyF	Function unknown	*A. longum*	WP_004098914.1	This study
YviE (DUF6470)	Function unknown	*B. subtilis*	WP_003243053.1	This study
DUF1217	Function unknown	*S. meliloti*	WP_026029868.1	This study
DUF327	Function unknown	*Clostridioides difficile*	WP_003436052.1	This study

## Data Availability

All MSAs, trees, homologous sequence sets, all the intermediate files and plots associated with our pipeline are available at Zenodo repository. All associated custom codes are available at our GitHub repository. Custom tools necessary to generate Figures 2–4 are available in our database at flagelladb.org. These resources provide full transparency and reproducibility for all computational steps described in this study. GitHub: https://github.com/Jouline-Lab/LBCA_Flagellum Zenodo: https://zenodo.org/records/20614833
