# Supplementary material for: The last bacterial common ancestor encoded a complex flagellum
Source: bioRxiv. 2026 Jun 12:2026.06.11.731707. Preprint. [Version 1] doi: 10.64898/2026.06.11.731707 (PMC13277936; doi:10.64898/2026.06.11.731707)
Supplement: Supplementary file 1 [file media-1.pdf]

SUPPLEMENTARY INFORMATION

The last bacterial common ancestor encoded a complex flagellum

Berkay Selçuk[1#], Ekaterina P. Andrianova[2,3#], Morgan Beeby[4], Daniel B. Kearns[5], Marc Erhardt[6,7], Igor B. Zhulin[1,2,3*]

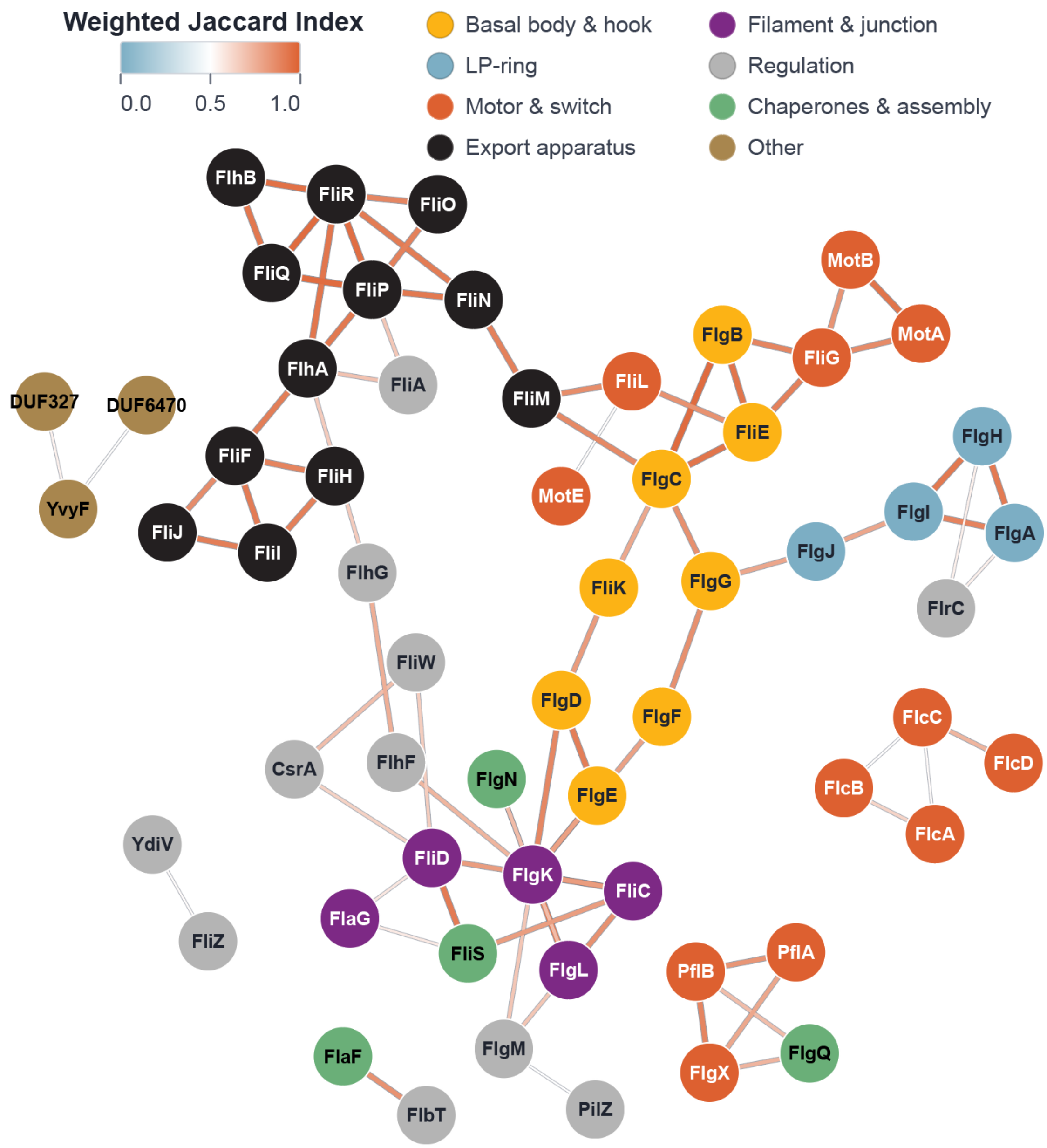

Figure S1: Network representation of family-weighted co-occurrence among flagellar genes. Force-directed network visualization of the pairwise family-weighted Jaccard similarity matrix shown in Figure 2d. Each node represents a flagellar gene and is colored according to its functional category. Edges connect gene pairs with a family-weighted Jaccard similarity >0.5, and edge color indicates similarity strength according to the scale shown. To highlight the strongest co-occurrence relationships while reducing network complexity, only the two highest-scoring connections were retained for each gene. Genes lacking any retained connections were omitted from the network.

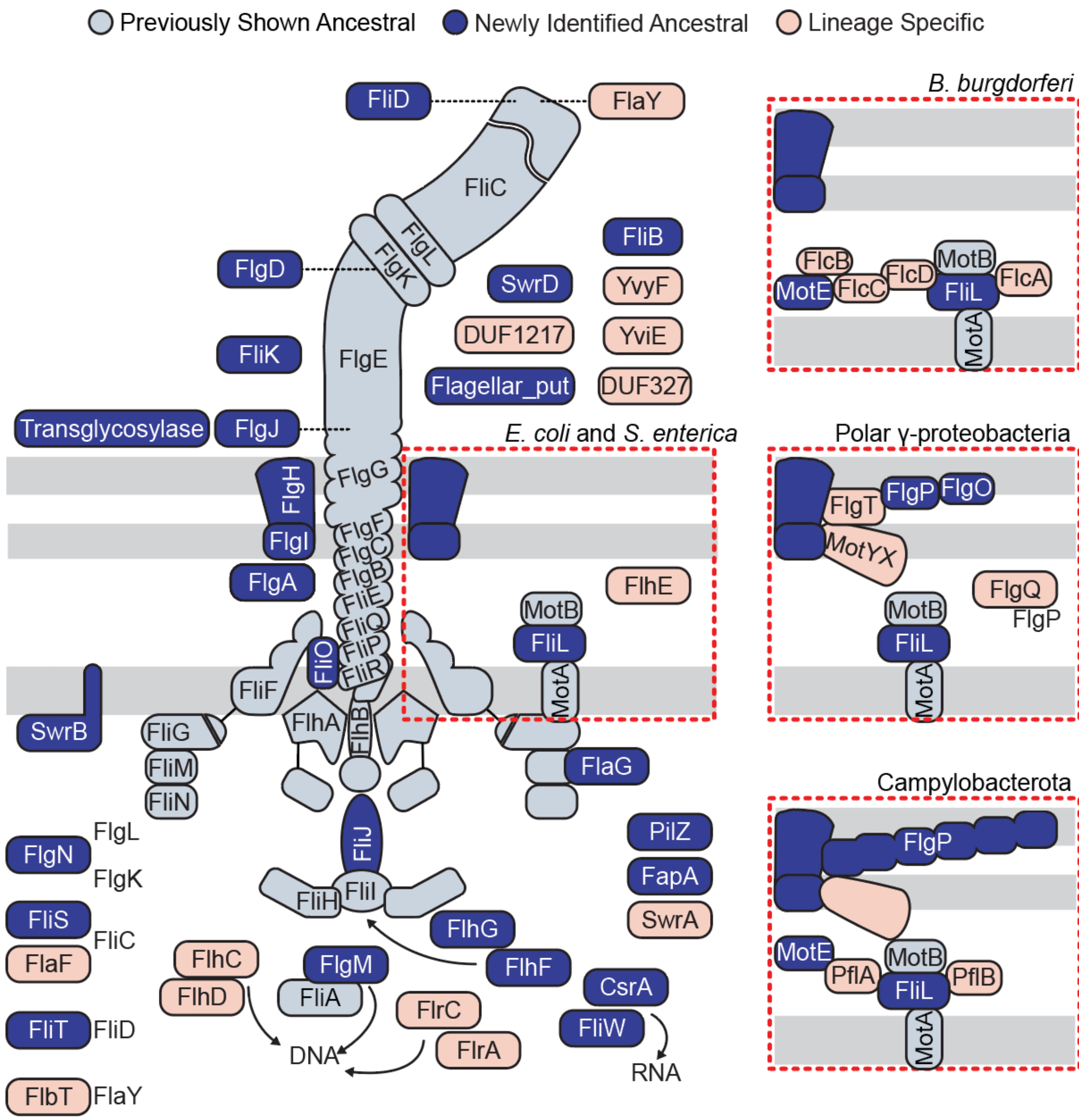

Figure S2. Reconstructed ancestral bacterial flagellum and lineage-specific flagellum-associated proteins. Schematic representation of the flagellar system inferred to have been present in the Last Bacterial Common Ancestor (LBCA). Components previously proposed to be ancestral are shown in light blue, whereas additional ancestral proteins identified in this study are shown in dark blue. Proteins inferred to have been acquired later during the diversification of bacterial lineages are shown in pink. Insets illustrate representative lineage-specific additions associated with the flagella of *Escherichia coli* and *Salmonella enterica*, spirochetes (*Borrelia burgdorferi*), γ-proteobacteria, and Campylobacterota.
